# Supplementary material for: Validity of constructed-response situational judgment tests in training programs for the health professions: A systematic review and meta-analysis protocol
Source: PLoS One. 2023 Jan 26;18(1):e0280493. doi: 10.1371/journal.pone.0280493 (PMC9879421; doi:10.1371/journal.pone.0280493)
Supplement: S1 File — (DOCX) [file pone.0280493.s002.docx]

## **Supporting Information**

## **S1 Table 1.** **Draft MEDLINE search strategy**

Our search strategy is composed of two elements: population (health professions education) and instrument (situational judgement test). We will not use outcome in our search strategy to avoid a very specific strategy that would limit the number of obtained papers.

| # | **Searches** |
| --- | --- |
| 1 | exp Schools, Health Occupations/ or exp Students, Health Occupations/ or Education, Medical/ or Education, Medical, Undergraduate/ or Education, Medical, Graduate/ or Students, Medical/ or Education, Dental/ or Education, Dental, Graduate/ or Education, Nursing/ or Education, Nursing, Baccalaureate/ or Education, Nursing, Graduate/ or Education, Pharmacy, Graduate/ or Students, Pharmacy/ or Education, Pharmacy/ or Education, Professional/ or Education, Veterinary/ |
| 2 | ((audiolog* or chiropract* or clinical or dental or dentistry or dietitian* or dietetic* or health profession* or kinesiolog* or massage therap* or medical or medicine or medical technol* or midwi* or music therap* or nursing or nutrition* or orthopt* or occupation* therap* or orthotis* or pharmac* or physician* assistant* or physical therap* or physiotherap* or phlebotom* or podiatrist* or prosthetist* or psycholog* or public health or radiation therap* or radio* or respiratory therap* or sonograph* or social worker* or speech therap* or veterinar*) adj2 (student* or trainee* or learner* or resident* or school* or program* or training or education* or curricul* or undergraduate* or postgraduate* or residen*)).ti,ab,kf. |
| 3 | 1 or 2 |
| 4 | (situational judgement test* or situational-judgement test* or SJT* or situational judgment test* or situational-judgment test*).mp. |
| 5 | exp social competence/ |
| 6 | exp personality/ |
| 7 | (non-cognit* or noncognit* or non-clinical or nonclinical or non-academic or emotional intelligen* or EI or social intelligen*).ti,ab,kf. |
| 8 | ((person* adj1 (attribute* or qualit* or skill* or characteristic*)) or (interperson* adj1 (attribute* or qualit* or skill* or characteristic*)) or (profession* adj1 (attribute* or qualit* or skill* or characteristic*)) or (soci* adj1 (attribute* or qualit* or skill* or characteristic*))).ti,ab,kf. |
| 9 | or/5-8 |
| 10 | (admi* or appl* or select* or evaluat* or test* or recruit* or assess* or matricul*).ti,kf. |
| 11 | 9 and 10 |
| 12 | 4 or 11 |
| 13 | (open or constructed or short or free or descriptive or questionnaire or audio or video or webcam or record* or audiovisual).ti,ab,kf. |
| 14 | 12 and 13 |
| 15 | 3 and 14 |

**S2 Table 2. Draft data extraction form**

| Details of citation | ID Number |
| --- | --- |
|  | Title |
|  | Authors |
|  | Publication Year |
|  | Language |
|  | Type (e.g., journal article, unpublished report) |
|  | Source (e.g., electronic search, grey literature, contact) |
| Details of study | Aims and objectives |
|  | Design (e.g., cross sectional, cohort, case control) |
|  | Setting (country, university, department) |
|  | Level of training (undergraduate, postgraduate) |
|  | Profession (e.g., medicine, nursing) |
|  | Phase (preclinical, clinical) (if applicable) |
|  | Cohort year* |
|  | Number and demographic characteristics of participants (e.g., age, gender, race) |
|  | Sampling method (if applicable) |
|  | Duration and follow up (if applicable)  Publication status  Type of predictive validity (concurrent or subsequent administration of the other measures) |
| Instruments** | Name of the tool |
|  | Assessment type (e.g., written, interview, OSCE, direct observation in the workplace) |
|  | Purpose (e.g., admission, in training formative, in training summative, license exam) |
|  | Content and construct (i.e., domains, aspects, competencies) |
|  | Structure (e.g., duration of the exam, number of items or stations) |
|  | Rating (e.g., number of raters, type of raters, training for raters) |
|  | Scoring, standard setting, and reporting system |
|  | Cost estimation |
|  | Any considerations (e.g., how dropout or incomplete follow-up have been addressed) |
| Key Findings | Outcomes measured (e.g., pass rate, distribution of scores, correlations, reliability) |
|  | Results (including the results for each subgroup) |
|  | Conclusion |

*If more than one year is included, details will be coded for each year separately if unique data are available.

** If more than one instrument has been used in one study, details for each of them will be coded separately.
