# Supplementary material for: Validity of constructed-response situational judgment tests in training programs for the health professions: A systematic review and meta-analysis protocol
Source: PLoS One. 2023 Jan 26;18(1):e0280493. doi: 10.1371/journal.pone.0280493 (PMC9879421; doi:10.1371/journal.pone.0280493)
Supplement: Attachment — Submitted filename: Response to Reviewers - Final.docx [file pone.0280493.s003.docx]

|  | Journal Requirements | Responses |  |
| --- | --- | --- | --- |
|  | 1. Please ensure that your manuscript meets PLOS ONE's style requirements, including those for file naming. The PLOS ONE style templates can be found at  <https://journals.plos.org/plosone/s/file?id=wjVg/PLOSOne_formatting_sample_main_body.pdf> and  <https://journals.plos.org/plosone/s/file?id=ba62/PLOSOne_formatting_sample_title_authors_affiliations.pdf>  2. Thank you for stating the following in the Competing Interests section:  YSP and AHS have no disclosures to declare. SMH, JLH, XP, and AM disclose they are salaried employees of Altus Assessments which administers a situational judgment test called Casper. The authors receive no reimbursements, fees, or funding related to this study or its outcomes.  Please confirm that this does not alter your adherence to all PLOS ONE policies on sharing data and materials, by including the following statement: "This does not alter our adherence to PLOS ONE policies on sharing data and materials.” (as detailed online in our guide for authors <http://journals.plos.org/plosone/s/competing-interests>). If there are restrictions on sharing of data and/or materials, please state these. Please note that we cannot proceed with consideration of your article until this information has been declared.  Please include your updated Competing Interests statement in your cover letter; we will change the online submission form on your behalf.  3. We note that you have referenced (ie. Bewick et al. [5]) which has currently not yet been accepted for publication. Please remove this from your References and amend this to state in the body of your manuscript: (ie “Bewick et al. [Unpublished]”) as detailed online in our guide for authors  <http://journals.plos.org/plosone/s/submission-guidelines#loc-reference-style>  4. Please include captions for your Supporting Information files at the end of your manuscript, and update any in-text citations to match accordingly. Please see our Supporting Information guidelines for more information: <http://journals.plos.org/plosone/s/supporting-information>. | 1. Many thanks for the links and resources. We have carefully updated the format of the manuscript to ensure it conforms with the journal’s style. This includes file naming, author bylines and affiliations, referencing, and citing the tables.  2. We read the resources you provided and also the journal’s guidelines regarding [Data Availability](https://journals.plos.org/plosone/s/competing-interests#loc-what-represents-a-competing-interest), and will be willing to add a sentence to clarify the influence of our employment on our ability to share the data. However, we are wondering what type of data is being exactly discussed here. Since this manuscript is the protocol of our planned study in which we have only indicated the rationale and methods of the study; no data have been collected or analyzed yet. When in the future, after completing the systematic review, we submit our full paper with the findings, we will make sure to include and declare any restrictions that we may have. Please advise if further action is required.  3. We have double checked to make sure that all references are included properly both in the text and in the reference list.  4. We updated both the captions and in-text citations of the Supporting Information files. |  |
|  | Reviewer #1 | Response | Page and Line |
| 1 | I appreciated the clarity and detail in the protocol provided. My primary questions are with regard to feasibility of a meta-analysis on this topic.  There are few published empirical studies or dissertations on constructed response SJTs (my quick search identified less than 25) across any setting and even fewer that are in Health Professionals Education programs. Within that, finding multiple studies that look at the same instrument or that look at the same outcome in order to look at cumulative effects seems highly unlikely to me. | Thank you for your comment on the feasibility of the proposed meta-analysis. We have addressed your helpful point by expanding and clarifying our definition of predictive validity to include studies that investigate the relation between SJTs and concurrent and future measures. This approach is better aligned with Messick’s unified validity framework which emphasizes a uniform conceptualization of validity evidence including 5 typical sources: content, response process, relations to other variables, internal structure, and consequences. In our review, we are going to focus on ‘relations to other variables’. Messick’s unified validity framework is endorsed by the Standards for Educational and Psychological Testing (AERA, APA, & NCME, 2014), which represents the consensus standard for validity and measurement communities in the field. In particular, ‘relations to other variables’ encompasses prior traditional concepts into a more modern source of validity evidence, including predictive and concurrent forms of validity evidence.  We have also clarified in the manuscript that we do not require instruments or outcomes to be the same to look at effects across studies. These changes will yield a sufficient number of papers to include in our systematic review and meta-analysis.    We recognize the reviewer’s concern regarding the potentially few published empirical studies or dissertations on the topic. We acknowledge that published research on constructed-response SJTs has historically been limited for meta-analyses but note that constructed-response SJTs have increased in popularity in recent years and there is a growing body of evidence suggesting their potential benefits relative to closed-response SJTs. Further, admissions leaders working in this subject area tend to have technical reports or quality assurance reports that they do not typically aim to publish in academic journals but use for the purposes of evaluating admission processes.  Our systematic review will provide a timely evaluation of both published evidence and grey literature that exists for the validity of constructed response SJTs and highlight potential areas for future research. It is for these reasons that we present that a systematic review and meta-analysis on constructed-response SJTs is particularly needed at this time. | Page 3-4, lines 58-71  &  Page 6, lines 125-131  Page 8, lines 174-175 |
| 2 | A second feasibility issue is with regard to the strategy for assessing moderated validity (i.e., differences in predictive validity by subgroup). Such comparisons would require having correlations between predictors and outcomes reported separately by subgroup -- the strategy outlined in the protocol suggests such a split would be possible, but it is rare to see this type of information reported in studies (published or unpublished) because of small Ns. Thus, it does not seem addressing that key question is feasible. | Thanks for pointing out this issue. We acknowledge that there are limited studies examining correlations between SJT scores and other measures reported separately by subgroup. As such we have clarified that this analysis is a secondary goal rather than the primary one. We would like to keep this as an objective given that accessing the potential moderated validity by subgroups is an important issue in medical education (assuming predictions are invariant across subgroups), and it would empirically demonstrate the diversity of validity from an equity perspective. The issue of differential predictive associations between subgroups is known as ‘predictive bias’ (Standards for Educational and Psychological Testing; AERA, APA, NCME &, 2014). From the existing literature, we don’t know if the validity evidence could be generalizable across demographic subgroups (e.g., gender, race, etc.) and this issue needs to be investigated further. We have modified our second question to “Depending on the data availability, the present systematic review will also look into the secondary question: to what extent are the relations…”.  If such information is not available, we will contact authors of the original reports and ask for data sets to calculate these splits ourselves. Given the importance of these questions in the medical education assessments community and the sparsity of their presentation, it will be particularly important to highlight it as a methodological gap across the literature. | Page 5 & 6, lines 111-123 |
| 3 | It seems that the key predictive validity questions would be focused on outcomes such as performance in classes, graduation rates, etc... Thus, it was unclear to me why looking at a variety of other types of assessments (e.g, personality measures) would be considered an examination of predictive validity rather than convergent validity. Further, because such assessments may be focused on specific constructs that are not those assessed by a given SJT it is unclear what such data will indicate. Perhaps an analysis that focuses on SJTs designed to measure certain attributes to measures designed to measure those same attributes would be a better focus. | Thanks for the comments and recommendations. As noted above (in response to comment #1), we have decided to extend the scope of the review to explore a general predictive validity of constructed-response SJTs, including both concurrent and future measures.  As noted earlier, we will include papers that investigated the relationships between constructed-response SJTs and other measures expected to evaluate similar attributes, either administered concurrently or subsequently. In this way, we will be able to include more papers in our review. Also, including both concurrent and future variables and providing information on a more general validity has significant practical relevance. Admission committees use constructed-SJTs to inform their selection process. By looking at the concurrent validity of constructed-SJTs, we will be able to inform admission committees on how they relate to concurrent admission evaluations such as Interviews and MMIs. Further, by looking at the predictive validity of constructed-SJTs, we will be able to provide insights for health professions educators and admission committees to understand relationships between constructed-SJTs and the existing evaluations within their program. | Page 3-4, lines 58-71  &  Page 6, lines 125-131 |
| 4 | It was surprising to see the note that SJTs have acceptable internal consistency as a common critique of SJTs are that they are multidimensional at the item level and often do not have high internal consistency. | We thank the reviewer for the comment. We have modified the sentence about acceptable internal consistency to the following:    “One meta-analysis showed that SJTs have an internal consistency coefficient that ranged from 0.43 to 0.94 ([McDaniel et al., 2001](https://pubmed.ncbi.nlm.nih.gov/11519656/); see also [Catano et al., 2012](https://onlinelibrary.wiley.com/doi/abs/10.1111/j.1468-2389.2012.00604.x)). However, it is often argued that lower internal consistency is found for SJTs because they commonly have a heterogenous structure representing many different factors ([Catano et al., 2012](https://onlinelibrary.wiley.com/doi/abs/10.1111/j.1468-2389.2012.00604.x); [Tiffin et al., 2019](https://pubmed.ncbi.nlm.nih.gov/31872483/)).” | Page 3, lines 58-66 |
| 5 | One question regarding constructed responses to assessments are the concerns over how scored -- for example, an oral SJT with constructed responses is akin to a situational interview. It seems looking at dimensions of scoring and who the assessors/raters are would be important aspects to code. | Thank you for this suggestion, but please note that Rating and Scoring dimensions are included in the data extraction form (Please see Appendix 2). | Appendix 2 |
|  | Reviewer #2 | |  |
| 1 | I think that meta-analyzing constructed-response SJTs is a worthwhile goal and I would enjoy reading that meta-analysis.  However, I think that to maximize the utility of findings for practitioners, they would need to know more than simply the average predictive validity of constructed-response (C-R) SJTs for selection into Health Professions Education. Based on the broader research on SJTs, we know that this is a method of measurement whose validity can vary greatly as a function of various methodological choices. So I'd expect a practitioner to want to know what methodological choices they should make when designing their C-R SJT in order to maximize its validity. In the case that the average validity is actually not very good, it would be especially important to be able to explain what makes some C-R SJTs more effective than others, rather than the take-home message ending up that these tools are not very useful.  As such, I would recommend that the authors pull all the available studies on C-R SJTs (within Health Professions or even more broadly than that) and determine potential moderators of validity to code and examine in their meta-analysis. The authors can look to the broader SJT research (outside of the Health Professions) for the types of methodological moderators other meta-analyses/reviews have considered (e.g., Campion, Ployhart, & MacKenzie, 2014; Christian, Edwards, & Bradley, 2010; McDaniel, Morgeson, Finnegan, Campion, & Braverman, 2001). While some moderators considered in those studies may not translate to C-R SJTs, others certainly do.  Related to the above, one potential moderator of validity I'd like to highlight is the validity study design (concurrent vs predictive). The authors indicated that they only intend to examine predictive validity designs, but that seems unnecessarily restrictive to me. I don't know about the Health Professions Education field in particular, but from the broader research on SJTs, do know that C-R SJTs constitute a small minority of SJTs in general, and predictive validity studies--as a much less common validity study design than concurrent--further restrict the possible sample of studies to include in a meta-analysis.  As a side note, from the authors' description of their plans, it was not entirely clear to me that they definitely plan to only examine predictive validity studies. For example, I did not understand this sentence (lines 137-138): "The measures may be administered at any point throughout the trainee's education to the completion of an SJT." And at the end of that same paragraph, this also doesn't sound like a predictive validity design: "The purpose of using the SJT may be for ... evaluation of students’ performance after admission."  Also, I would expect raters who score responses to C-R SJTs (e.g., their training) to also have validity-moderating potential. I noticed that the example of potential moderators I bring up are actually already reflected in the protocol for what the researchers planned to code about the articles they find (Appendix 2), but the data extraction form may need further refinement to capture anything additional about the research methodology that might be relevant. Again, I believe that the more validity moderators the authors can point to, the more practical utility their research will have. | Thank you so much for your encouraging feedback. Also, many thanks for your detailed comment and explanation. As suggested, we have taken a broader concept of validity (including both concurrent and future measures), conceptualized using Messick’s unified validity framework, as operationalized in the Standards for Educational and Psychological Testing (AERA, APA, & NCME, 2014). This change is aligned with the recommendations made by the first reviewer (comments #1 and #3). We have clarified this throughout the manuscript including by adding the definition and framework of validity to the introduction.    Given that SJT validity can vary greatly as a function of their design features, we will be collecting data on the potential moderators. A preliminary list of the potential moderators had been stated in the data extraction form (Appendix 2 - Instruments). As per your suggestion, we made modifications to capture additional features (e.g., publication status). For this purpose, we used the recommended references, including the paper by Campion et al. (2014).  This revision will let us discuss what makes some tools more effective than others. So, assessment teams will know their methodological choices when designing or selecting a SJT and can maximize the validity outcomes of their assessments. | Page 3-4, lines 58-71  &  Page 6, lines 125-131  Page 4, lines 78-83 & Appendix 2 |
| 2 | As a final note, it was unclear to me how the authors plan to examine the effect of possible publication bias (lower quality studies remaining in a file drawer) on their findings (lines 237-238). They may find these sources helpful:  Duval SJ, Tweedie RL. (2000). A non-parametric “trim and fill” method of accounting for publication bias in meta-analysis. Journal of the American Statistical Association, 95, 89–98.  Duval SJ, Tweedie RL. (2000). Trim and fill: A simple funnel plot-based method of testing and adjusting for publication bias in meta-analysis. Biometrics, 56, 276–284. | Thanks for pointing out this important issue. We would like to acknowledge that file drawer does not necessarily mean that studies are low quality (e.g., studies can be rejected from publication not because of their level of quality or rigor, but rather due to reasons such as journals’ preference for “statistically significant” findings; [Harrison, 2017](https://journals.sagepub.com/doi/10.1177/0149206314535438); [Simonsohn et al., 2014](https://pubmed.ncbi.nlm.nih.gov/26186117/)). Meanwhile, studies that end up published may have been more likely to be subject to questionable research practices such as p-hacking, HARKing, etc. ([Banks et al., 2016](https://link.springer.com/article/10.1007/s10869-016-9456-7); [O'Boyle et al., 2014](https://journals.sagepub.com/doi/abs/10.1177/0149206314527133); [Simonsohn et al., 2015](https://psycnet.apa.org/record/2015-53127-004); [Xie et al., 2021](https://link.springer.com/article/10.1007/s11948-021-00314-9))  The method we are considering for looking at publication bias is first to code publication status as a moderator (i.e., published vs unpublished). Then, depending on the representation of studies across quality and publication status, we will employ statistical methods for accounting for publication bias, as suggested. We are considering this, as suggested in the PRISMA guide under ‘meta-bias’. |  |
