# Supplementary material for: Validity of constructed-response situational judgment tests in training programs for the health professions: A systematic review and meta-analysis protocol
Source: PLoS One. 2023 Jan 26;18(1):e0280493. doi: 10.1371/journal.pone.0280493 (PMC9879421; doi:10.1371/journal.pone.0280493)
Supplement: Attachment — Submitted filename: PONE-D-22-08111R1 - Response to Reviewers - 20221202.docx [file pone.0280493.s004.docx]

Dear Editor in Chief,

We would like to thank the reviewers for their considerate feedback and helpful suggestions, many of which have been implemented into the revised manuscript.

Clean and ‘tracked changes’ versions of the manuscript have been uploaded. We have also provided point-by-point responses to each reviewer’s remarks in the associated table. The line numbers in the table refer to line numbers in the *tracked changes* version.

We appreciate the opportunity to revise our manuscript and look forward to hearing from you.

Thank you,

| **#** | **Reviewer comment** | **Response** | *TC* **Line reference** |
| --- | --- | --- | --- |
| **Reviewer 1** | | | |
| 1 | While the author(s) did address comments, there was insufficient compelling evidence that there is sufficient literature available for a review | We appreciate the reviewer’s concern on this matter. We informally investigated the extent of literature available for a review. In the appendix the reviewer can find citations for 21 potentially relevant studies, each addressing validity of constructed-response SJTs in the health professions. These references have not undergone full-text review, however, they indicate that there have been at least 18 years of research on the topic to date (2004-2022) and that the topic is an active area of research.  In addition to published articles, in the manuscript we state that *“we will also contact academics and researchers and invite them to share raw or summary association information, whitepaper reports, technical quality assurance reports, or conference papers to be included in the study”.* For instance, we are aware that Casper, a constructed-response SJT, is used by [more than 300 professional health science programs,](https://takealtus.com/schools-and-programs/) and so we anticipate programs will provide additional summary association information or technical reports.  We are also confident in there being evidence for review because, from a practical perspective, health professions programs currently use constructed-response SJTs to make admissions and evaluation decisions. Programs often rely on anecdotal evidence or small associative investigations with in-program measures (both behavioral and curricular) to support the use of SJTs. Program administrators may acknowledge that the outcome measures they use for these decisions only partially address aspects of situational judgment or professionalism.  At the same time, they acknowledge that in clinical practice, situational judgment and professionalism interact with the individual’s academic knowledge and clinical skills. This is further reflected using outcome evaluation measures which are assessed by each program. Success, as defined by the health profession program, is indeed institute-specific and program measures commonly include academic knowledge, clinical skills, and professionalism items together (for example the Objective Structured Clinical Examinations (OSCEs). [Gromley 2011](https://www.ncbi.nlm.nih.gov/pmc/articles/PMC3605523/)).  To account for this current practice, measures that are described by the authors to address situational judgment or professionalism at least partially will be included in this review. Specifically, we will include studies measuring outcomes that have overlap with aspects measured by the implemented situational judgment test. For instance, most health professions governing bodies (e.g. [CanMEDS](https://www.royalcollege.ca/rcsite/canmeds/canmeds-framework-e), [AAMC](https://students-residents.aamc.org/applying-medical-school/article/core-competencies), [ACGME](https://knowledgeplus.nejm.org/blog/acgme-core-competencies-professionalism/), [PAEA](https://paeaonline.org/wp-content/uploads/2021/03/competencies-for-the-pa-profession-2020605.pdf)) define and describe aspects of professionalism, and the SJT may reference these descriptions or the aspects themselves. Examples of aspects include but are not limited to:   - [Interpersonal and Communication Skills](https://knowledgeplus.nejm.org/blog/acgme-core-competencies-interpersonal-and-communication-skills/) - “demonstrate the ability to effectively exchange information with patients, their families, and professional associates…” - [Leader](https://www.royalcollege.ca/rcsite/canmeds/framework/canmeds-role-leader-e) - “As Leaders, physicians engage with others to contribute to a vision of a high-quality health care system and take responsibility for the delivery of excellent patient care through their activities as clinicians, administrators, scholars, or teachers.” - [Reliability and Dependability](https://students-residents.aamc.org/applying-medical-school/article/core-competencies) - “Consistently fulfills obligations in a timely and satisfactory manner; takes responsibility for personal actions and performance.”   We believe this approach aligns with real-life practice. As such we have added the following methodological improvements to the manuscript:  *“Instruments: We will include studies reporting on the association of a constructed-response SJT with at least one other measure that, partially or completely, aims to assess personal, interpersonal, or professional attributes. To ensure that the SJT and the outcome measure at least partially assess the same or similar underlying construct, the intended aspects of evaluation will be recorded. For instance, descriptions from both the SJT and the outcome measure will need to reference at least one of the same aspects of professionalism or situational judgment. These aspects may be components of professionalism definitions or descriptions from health professions governing bodies (e.g., CanMEDS, ACGME) and can include but are not limited to aspects such as interpersonal and communication skills, leadership, reliability and dependability. If there are no overlapping aspects between the SJT and the outcome measure, the study will be excluded.”*  *…*  *“ To improve reporting transparency and mitigate the risk of instrument heterogeneity, a coding scheme will be implemented to organize measures based on their degree of alignment to the underlying construct or aspects assessed by the constructed-response SJT. Results from instruments with high overlap, where the measure’s score is predominantly derived from aspects assessed by the SJT, will be grouped together. Conversely, results from instruments with low overlap will be grouped together. That is, where the outcome measure’s score is predominantly derived from aspects not assessed in the SJT (e.g., academic, procedural knowledge or clinical skills).”*  Given the results of our informal investigation and the extent to which constructed-response SJTs are already being incorporated into health professions programs, we believe it is important and feasible to synthesize the evidence and provide direction to continue improving the implementation of these tools. | 230  177  305 |
| **Reviewer 2** | | | |
| 1 | In using Messick's validity framework, I think the authors should also adjust their terminology and refer to the test's "validity" or "construct validity," as opposed to its "predictive validity," which has a narrower definition and can be confusing to readers as referring to study designs where the SJT predicts an outcome measure separated in time from the SJT (predictor). | Thank you to the reviewer for the helpful feedback and considerate responses.  As suggested, to align with Messick’s framework, we have revised the terminology throughout the manuscript and refer to *validity* in place of *predictive validity*. The context and focus of validity with respect to Messick’s framework is articulated by the following paragraph, which can be found at the end of the introduction:  *“To be aligned with modern perspectives on validity, we will frame our study using Messick’s framework that emphasizes a uniform conceptualization of validity evidence including content, response process, relations to other variables, internal structure, and consequences, as operationalized in the Standards for Educational and Psychological Testing [24]. In this study, we will synthesize the collected evidence regarding ‘relations to other variables’. In this sense, we will use a definition of the validity that includes the relations to both concurrent and subsequent measures.”* | 130 |
| 2 | I don't think the other reviewer's concern has been adequately addressed regarding the authors being able to find multiple studies that look at the same instrument or that look at the same outcome in order to be able to consider cumulative effects.  The authors say this is not a requirement for them to be able to do their analyses, but I did not see a rationale in the response to reviewers or the revised proposal. It's unclear to me what the analyses would indicate without attending to the constructs assessed by the instruments involved. | We thank the reviewer for their feedback. We agree that finding multiple studies looking at the same instrument is a potential concern. We also acknowledge that the previous version of the manuscript did not adequately address the rationale for continuing or steps to mitigate this risk. Despite an expected limited number of studies investigating identical instruments, we consider this investigation feasible and warranted for the following reasons:  First, from a practical perspective, health professions programs currently use constructed-response SJTs to make admissions and evaluation decisions. Programs often rely on anecdotal evidence or small associative investigations with in-program measures (both behavioral and curricular) to support the use of SJTs. Program administrators may acknowledge that the outcome measures they use for these decisions only partially address aspects of situational judgment or professionalism.  At the same time, they acknowledge that in clinical practice, situational judgment and professionalism interact with the individual’s academic knowledge and clinical skills. This is further reflected using outcome evaluation measures which are assessed by each program. Success, as defined by the health profession program, is indeed institute-specific and program measures commonly include academic knowledge, clinical skills, and professionalism items together (for example the Objective Structured Clinical Examinations (OSCEs). [Gromley 2011](https://www.ncbi.nlm.nih.gov/pmc/articles/PMC3605523/)).  To account for this current practice, measures that are described by the authors to address situational judgment or professionalism at least partially will be included in this review. Specifically, we will include studies measuring outcomes that at least partially overlap with aspects measured by the implemented situational judgment test. For instance, most health professions governing bodies (e.g. [CanMEDS](https://www.royalcollege.ca/rcsite/canmeds/canmeds-framework-e), [AAMC](https://students-residents.aamc.org/applying-medical-school/article/core-competencies), [ACGME](https://knowledgeplus.nejm.org/blog/acgme-core-competencies-professionalism/), [PAEA](https://paeaonline.org/wp-content/uploads/2021/03/competencies-for-the-pa-profession-2020605.pdf)) define and describe aspects of professionalism, and the SJT may reference these descriptions or the aspects themselves. Examples of aspects include but are not limited to:   - [Interpersonal and Communication Skills](https://knowledgeplus.nejm.org/blog/acgme-core-competencies-interpersonal-and-communication-skills/) - “demonstrate the ability to effectively exchange information with patients, their families, and professional associates…” - [Leader](https://www.royalcollege.ca/rcsite/canmeds/framework/canmeds-role-leader-e) - “As Leaders, physicians engage with others to contribute to a vision of a high-quality health care system and take responsibility for the delivery of excellent patient care through their activities as clinicians, administrators, scholars, or teachers.” - [Reliability and Dependability](https://students-residents.aamc.org/applying-medical-school/article/core-competencies) - “Consistently fulfills obligations in a timely and satisfactory manner; takes responsibility for personal actions and performance.”   Second, from a feasibility perspective, the approach for evaluating effects across multiple instruments or scales is addressed in the [PRISMA 2020 explanation and elaboration: updated guidance and exemplars for reporting systematic reviews](https://www.bmj.com/content/372/bmj.n160). Specifically, Item 12 discusses the capacity to re-express results to aid in interpreting the *“size of effect (such as minimally important difference; ranges for no/trivial, small, moderate, and large effects).”*  And importantly to *“consider providing justification for the choice of effect measure. For example, a standardized mean difference may have been chosen because multiple instruments or scales were used across studies to measure the same outcome domain (such as different instruments to assess depression).”*  As the reviewer highlights, the study’s outcome must have assessed the same or a similar construct. Synthesizing results from multiple measures can be advantageous where possible. When only identical measures are pooled it prohibits all the available evidence from being included. This in turn can also lead to imprecise estimates and few studies being included ([Hassan Murad et al 2019](https://www.ncbi.nlm.nih.gov/pmc/articles/PMC6890471/)).  To further mitigate the risk of multiple instruments, as part of the data extraction and synthesis process, the instruments will be coded into subgroups. The coding scheme will organize measures based on their degree of alignment to the underlying construct or aspects assessed by the implemented constructed-response SJT. An example of this coding scheme methodology can be seen in a similar review in the educational research domain ([Wisniewski et al 2020](https://www.frontiersin.org/articles/10.3389/fpsyg.2019.03087/full)).  The above rational and methodological improvements have been added to the manuscript methods as follows:  *“Instruments: We will include studies reporting on the association of a constructed-response SJT with at least one other measure that, partially or completely, aims to assess personal, interpersonal, or professional attributes. To ensure that the SJT and the outcome measure at least partially assess the same or similar underlying construct, the intended aspects of evaluation will be recorded. For instance, descriptions from both the SJT and the outcome measure will need to reference at least one of the same aspects of professionalism or situational judgment. These aspects may be components of professionalism definitions or descriptions from health professions governing bodies (e.g., CanMEDS, ACGME) and can include but are not limited to aspects such as interpersonal and communication skills, leadership, reliability and dependability. If there are no overlapping aspects between the SJT and the outcome measure, the study will be excluded.”*  *…*  *“To improve reporting transparency and mitigate the risk of instrument heterogeneity, a coding scheme will be implemented to organize measures based on their degree of alignment to the underlying construct or aspects assessed by the constructed-response SJT. Results from instruments with high overlap, where the measure’s score is predominantly derived from aspects assessed by the SJT, will be grouped together. Conversely, results from instruments with low overlap will be grouped together. That is, where the outcome measure’s score is predominantly derived from aspects not assessed in the SJT (e.g., academic, procedural knowledge or clinical skills).”*    In addition, to address the other reviewer’s concern, we provided the following additional information (also reported directly to the first reviewer in the first row of this table):  “n the appendix,appendix the reviewer can find citations for 21 potentially relevant studies, each addressing validity of constructed-response SJTs in the health professions. These references have not undergone full-text review. However, they indicate that there have been at least 18 years of research on the topic to date (2004-2022) and that the topic is an active area of research.  In addition to published articles, in the manuscript we state that *“we will also contact academics and researchers and invite them to share raw or summary association information, whitepaper reports, technical quality assurance reports, or conference papers to be included in the study”.* For instance, we are aware that Casper, a constructed-response SJT, is used by [more than 300 professional health science programs,](https://takealtus.com/schools-and-programs/) and so we anticipate programs will provide additional summary association information or technical reports.  Given the results of our informal investigation and the extent to which constructed-response SJTs are already being incorporated into health professions programs, we believe it is important and feasible to synthesize the evidence and provide direction to continue improving the implementation of these tools. | 177  305  230 |
| 3 | While the authors acknowledge in their responses that it is important to code potential moderators of validity in their review of the literature, there is no research question posed in the proposal intro to look at methodological moderators of validity as part of their analyses. | Thank you for pointing out this oversight. We indeed intend to investigate methodological moderators where possible, as indicated in the introduction when identifying the literature gaps. These methodological considerations will be coded during data extraction. To clarify the objective, the following phrase has been added at the end of the introduction:  *“Depending on the data availability, the present systematic review will also investigate the secondary questions:*   - *To what extent are the relations between constructed-response SJT scores and other measures of personal, interpersonal, and professional attributes assessed either concurrently or in the future moderated by demographic characteristics (e.g., gender, race, socioeconomic status, language) and methodological variables (e.g., test construction, study design, response instruction)?”* | 129 |
| **Reviewer 3** | | | |
| 1 | The term "health professions education programs" was a bit confusing to me at the beginning and made me think of programs that aim to train educators rather than health professionals. Thus, I suggest that the authors replace it with another more clear term like health sciences/professional training programs. | We apologize for the confusion experienced by the reviewer. We have clarified the setting at the beginning of the introduction to avoid this confusion for other readers (please see excerpt below).  *“Training programs for the health professions (e.g., medicine, nursing, physician assistants, physical and occupational therapy, etc.) have traditionally prioritized applicants’ academic knowledge and skills, such as their grade point average (GPA) and Medical College Admission Test (MCAT) scores as indicators of their future success [1].”*  The term *HPE* has also been replaced with “training programs for health profession programs” throughout. | 51 |
| 2 | The outcomes and the acceptable assessment tools that assess “other measures of personal, interpersonal, and professional attributes” warrant further discussions and clarifications because these are the tests against which your main test is being tested and needs to be validated. Do the authors have pre-specified inclusion criteria for these tests?  Is any comparison considered acceptable? I assume this issue can impact the validity of the final results because these measures will vary in their discriminative and diagnostic features. I think in this case, your study can also be formulated into a diagnostic question where these measures may represent the “reference standard”. The same case goes for clinical prediction rules which are similar to this study. | We thank the reviewer for bringing up this important point. Indeed, the variability in the outcome measures concerning personal, interpersonal, and professional attributes will impact our final results. The following sections have been added to the methods of the manuscript to further specify the inclusion criteria of acceptable instruments, and how they will be identified and reported.  *“Instruments: We will include studies reporting on the association of a constructed-response SJT with at least one other measure that, partially or completely, aims to assess personal, interpersonal, or professional attributes. To ensure that the SJT and the outcome measure at least partially assess the same or similar underlying construct, the intended aspects of evaluation will be recorded. For instance, descriptions from both the SJT and the outcome measure will need to reference at least one of the same aspects of professionalism or situational judgment. These aspects may be components of professionalism definitions or descriptions from health professions governing bodies (e.g., CanMEDS, ACGME) and can include but are not limited to aspects such as interpersonal and communication skills, leadership, reliability and dependability. If there are no overlapping aspects between the SJT and the outcome measure, the article will be excluded.”*  In addition, we have added a component to the data synthesis section to differentiate between measures based on how closely they align with the underlying construct assessed by the SJT used.  *“To improve reporting transparency and mitigate the risk of instrument heterogeneity, a coding scheme will be implemented to organize outcome measures based on their degree of alignment to the underlying construct assessed by the constructed-response SJT. Results from instruments with high overlap, where greater than half the measure’s score is derived from aspects assessed by the SJT, will be grouped together. Conversely, results from instruments with low overlap will be grouped together. That is, where greater than half the measure’s score is derived from aspects not assessed in the SJT (e.g., academic, procedural knowledge or clinical skills).”*  Finally, we have added to the outcome section language clarifying the anticipated analyses performed.  *“Outcomes: We will include studies that have reported the magnitude of relation between constructed-response SJT scores and other measures of personal, interpersonal, and professional attributes. These are expected to include univariate and multivariate techniques such as linear and logistic regression analyses, classification techniques, relative risk estimates (e.g., odds ratios) and risk predictions, similar to methods observed in evaluating clinical prediction rules (*[*Cowley et al 2019*](https://diagnprognres.biomedcentral.com/articles/10.1186/s41512-019-0060-y)*).”*  We agree and acknowledge that the differences in discriminative and diagnostic features between outcome measures will influence the results. However, health professions programs currently use constructed-response SJTs to make admissions and evaluation decisions. Programs often rely on anecdotal evidence or small associative investigations with in-program measures (both behavioral and curricular) to support the use of SJTs. Program administrators may acknowledge that the outcome measures they use for these decisions only partially address aspects of situational judgment or professionalism.  At the same time, they acknowledge that in clinical practice, situational judgment and professionalism interact with the individual’s academic knowledge and clinical skills. This is further reflected using outcome evaluation measures which are assessed by each program. Success, as defined by the health profession program, is indeed institute-specific and program measures commonly include academic knowledge, clinical skills, and professionalism items together (for example the Objective Structured Clinical Examinations (OSCEs). [Gromley 2011](https://www.ncbi.nlm.nih.gov/pmc/articles/PMC3605523/)).  Given the extent to which constructed-response SJTs are already being incorporated into health professions programs and the modifications suggested above to improve the study, we believe it is feasible and pertinent to synthesize the evidence and provide direction to continue improving the implementation of these tools. | 177  305  202 |
| 3 | While the currently suggested risk of bias tool seems appropriate and meets the general standards, I suggest that the authors also consider the PROBAST (Prediction model Risk Of Bias Assessment Tool) which may be more helpful for their question which is a mixture of diagnosis and prognosis. | We would like to thank the reviewer for this suggestion. We have investigated PROBAST as compared to the current solution.  While promising, there were a few implementation notes on the [PROBAST Explanation and Elaboration](https://www.probast.org/wp-content/uploads/2020/02/aime201901010-m181377.pdf) article. First, the authors indicate *“PROBAST is not designed to assess predictor finding studies, where the aim of multivariable modeling is to identify predictors associated with the outcome rather than to develop a model for individualized predictions (16, 68, 69). The QUIPS (Quality in Prognosis*  *Studies) tool has been developed for assessment of*  *bias in these studies (70).”* We expect most articles will not investigate individualized predictions but in fact look at identifying the association between predictors (the SJT) and outcomes. Second, while it may not be a strict requirement for PROBAST, we note that it is developed specifically for multivariable models. We intend to include studies that complete univariate analysis and would not want to exclude for this reason. Finally, upon re-investigating the applicability of the Quality In Prognosis Studies (QUIPS) tool we would note that the tool was also recently used in another systematic review and meta-analysis of SJTs, which closely matches the format of the current study ([Webster et al 2020](https://onlinelibrary.wiley.com/doi/full/10.1111/medu.14201)). We found their risk of bias assessment was clear and applicable to the instruments and outcomes within this field.  We are happy the reviewer agrees that the current risk of bias tool seems appropriate and meets general standards and thank them for introducing us to other potentially helpful resources. For the reasons above, we have elected to maintain the QUIPS tools in the protocol. |  |

# Appendix - Potential References

## Journal articles

1. Alkhuzaee, F. S., Ali, M., Goh, K. W., Al-Worafi, Y. M., & Ming, L. C. (2022). Situational judgment using ethical reasoning in Saudi undergraduate pharmacy students. BMC medical ethics, 23(1), 1-9.
2. Barber, C., Burgess, R., Mountjoy, M., Whyte, R., Vanstone, M., & Grierson, L. (2022). Associations between admissions factors and the need for remediation. Advances in Health Sciences Education, 27(2), 475-489.
3. de Visser M, Fluit C, Cohen-Schotanus J, Laan R. The effects of a non-cognitive versus cognitive admission procedure within cohorts in one medical school. Adv Health Sci Educ Theory Pract. 2018 Mar;23(1):187-200. doi: 10.1007/s10459-017-9782-1. Epub 2017 Jun 10. PMID: 28601913; PMCID: PMC5801390.
4. Dore, K. L., Reiter, H. I., Kreuger, S., & Norman, G. R. (2017). CASPer, an online pre-interview screen for personal/professional characteristics: prediction of national licensure scores. Advances in Health Sciences Education, 22(2), 327-336.
5. Dore, K. L., Reiter, H. I., Eva, K. W., Krueger, S., Scriven, E., Siu, E., ... & Norman, G. R. (2009). Extending the interview to all medical school candidates—Computer-Based Multiple Sample Evaluation of Noncognitive Skills (CMSENS). Academic Medicine, 84(10), S9-S12.
6. Groene, O. R., Knorr, M., Vogel, D., Hild, C., & Hampe, W. (2022). Reliability and validity of new online selection tests for midwifery students. Midwifery, 106, 103245.
7. Juster, F. R., Baum, R. C., Zou, C., Risucci, D., Ly, A., Reiter, H, et al. Addressing the diversity-validity dilemma using situational judgment tests. Acad Med. 2019;94(8):1197–203.
8. Shipper, E. S., Mazer, L. M., Merrell, S. B., Lin, D. T., Lau, J. N., & Melcher, M. L. (2017). Pilot evaluation of the computer-based assessment for sampling personal characteristics test. Journal of Surgical Research, 215, 211-218.
9. Yingling, S., Park, Y. S., Curry, R. H., Monson, V., & Girotti, J. (2018). Beyond cognitive measures: Empirical evidence supporting holistic medical school admissions practices and professional identity formation. MedEdPublish, 7.
10. Zou, C., McConnell, M., Leddy, J., Antonacci, P., & Lemay, G. (2018). Comparison of the English and French versions of the CASPer® Test in a bilingual population. MedEdPublish, 7(281), 281.

## Conference papers and presentations

11. Burgess, R., Barber, C., Mountjoy, M., Whyte, R., Vanstone, M., & Grierson, L. (2020). Evaluating the validity evidence of admissions and medical school performance variables on student outcomes at McMaster University (Hamilton, Canada). [Association for Medical Education in Europe](https://amee.assetbank-server.com/assetbank-amee/action/browseItems?categoryId=562&categoryTypeId=2&cachedCriteria=1).
12. Fanning, S. L., Proscia-Lieto, T., Buskey, T. J., Clayton, K. E., Rose, F., Toplan, S., Burke, D. A., Dady, N., Cammarata, J., & Forstein, D. A. (2020). Incorporation of CASPer Online Situational Judgment Test to Enhance the Holistic Admissions Process. The Journal of the American Osteopathic Association, 120(3), e16–e17. <https://doi.org/10.7556/jaoa.2020.035>
13. Hulsman, R. L., Mollema, E. D., Hoos, A. M., De Haes, J. C. J. M., & Donnison-Speijer, J. D. (2004). Assessment of medical communication skills by computer: assessment method and student experiences. Medical Education, 38(8), 813-824.
14. Keene J. R., Novack, J., Baldwin, M., Mattocks, P., Randolph-Habecker, J., Whisler, J., Hunter Chase, L., Case, C., Gothard, M. D., & Enns, L. (2020) SJT testing using CASPer: Predicting commitment to underserved medicine [paper presentation]. From: Educating Leaders 2020: [The AACOM Annual Conference, Virtual.](https://cslide-us.ctimeetingtech.com/aacom2020/attendee/eposter/poster/29)
15. Leduc, J., Gagnon, R., Akim, V., Rioux, R., Bégin, P., Loye, N., Béland, S., Renaud, J., Hudon, C., Bourget, M., Ouellet, A., Gauthier, I., Samandi, S., Chetrit, E., Razack, S., Hooton, K., & Bourdy, C. (2020). Associations between CASPer scores and sociodemographic characteristics of applicants: preliminary findings from a multicenter study in Quebec. [Canadian Conference on Medical Education](https://events.decorporate.ca/CCME2020/abstract/submission-summary.php?y=cGFwZXJJZD01MTYwMiZyZW1vdmVWYWxpZGF0aW9uPXllcw==)
16. Parker-Newlyn (2020) “CASPer down under” - Piloting an online situational judgment test for medical school selection in Australia. [Canadian Conference on Medical Education](https://events.decorporate.ca/CCME2020/abstract/submission-summary.php?y=cGFwZXJJZD01MTQ0OCZyZW1vdmVWYWxpZGF0aW9uPXllcw==)
17. Roduta-Roberts & M. Alves C. (2021). Collecting validity evidence for CASPer: Predicting OSCE and fieldwork performance within an occupational therapy program. [Presentation]. American Educational Research Association.
18. Roduta-Roberts, M., Alves, C., & Chen, F. (2022). Validity evidence for the use of Casper in admissions decisions. [Presentation]. Canadian Association of Occupational Therapists.
19. Saxena, A., Desanghere, L., Dore, K.,& Reiter, H. (2021). Incorporating Situational Judgment Tests Into Postgraduate Medical Education Admissions: Examining Educational and Organizational Outcomes. Academic Medicine: 96(11S), S203-S204. [https://do.org/10.1097/ACM.0000000000004280](https://doi.org/10.1097/ACM.0000000000004280)
20. Woodson, M., Hussin, A., & MacIntosh, A. (2022). Evaluating medical school admissions screening measures to promote diversity while relating to future clerkship performance [paper presentation]. [American Education Research Association](https://events.decorporate.ca/CCME2021/abstract/submission-summary.php?y=cGFwZXJJZD02MDYwMSZsYW5ndWFnZUlkPTEwMA==).
21. Yan, Lemay, Wood (2022) Does Casper predict medical school performance? [Canadian Conference on Medical Education](https://events.decorporate.ca/CCME2022/abstract/submission-summary.php?y=cGFwZXJJZD02OTMwNCZsYW5ndWFnZUlkPTEwMA==).
